# Potential benefits of metformin and pioglitazone combination therapy via gut microbiota and metabolites in high-fat diet-fed mice

**DOI:** 10.3389/fphar.2022.1004617

**Published:** 2022-10-11

**Authors:** Dongmei Wang, Jieying Liu, Ling Zhong, Lu Ding, Qian Zhang, Miao Yu, Ming Li, Xinhua Xiao

**Affiliations:** ^1^ Department of Endocrinology, NHC Key Laboratory of Endocrinology, Peking Union Medical College Hospital, Peking Union Medical College and Chinese Academy of Medical Sciences, Beijing, China; ^2^ Department of Medical Research Center, Peking Union Medical College Hospital, Chinese Academy of Medical Sciences and Peking Union Medical College, Beijing, China

**Keywords:** pioglitazone, metformin, combination therapy, gut microbiota, metabolites

## Abstract

Metformin and pioglitazone monotherapy have been proven to alter gut microbiota in diabetes and obesity. The present study aimed to investigated whether the combined administration of pioglitazone and metformin achieved superior protective effects on high-fat diet (HFD)-fed obese mice and elucidated its molecular mechanism *via* the gut microbiota and its metabolites. C57BL/6 males were randomly divided into five groups: the control group, fed a normal control diet; the HFD group, fed an HFD; the metformin monotherapy group, fed an HFD and treated with metformin; the pioglitazone monotherapy group, fed an HFD and treated with pioglitazone; and the combination therapy group, fed an HFD and treated with metformin and pioglitazone combination therapy. The cecal contents were collected for 16S rDNA amplicon sequencing and untargeted metabolomics analysis. The results showed that the combination therapy of metformin and pioglitazone significantly improved insulin sensitivity and glucolipid metabolism in HFD-fed mice. Combination therapy markedly altered gut microbiota by increasing beneficial bacteria, such as *Bifidobacterium,* Christensenellaceae*_R-7_group, Faecalibacterium* and *Roseburia*, and decreasing harmful bacteria, such as *Oscillibacter* and *Eubacterium_xylanophilum_group*. Fecal metabolites were significantly changed in the combination therapy group, including a reduction in amino acid metabolism and augmentation of lipid metabolism, such as citrulline, sarcosine, D-glutamine, lipoxin A4, prostaglandin E2, stearidonic acid and lucidenic acid A. These results revealed that combined metformin and pioglitazone therapy had synergistic effects or at least have an additive effect on modifying gut microbiota and metabolites, closely associated with improved glucolipid metabolic parameters in HFD-fed mice, which provides novel evidence and promising targets for metformin and pioglitazone combination therapy in type 2 diabetes.

## 1 Introduction

Diabetes is one of the most common chronic diseases worldwide, and it imposes a true global burden of mortality and disability due to its complex complications (Zheng et al., 2018). The prevalence of diabetes in adults was estimated to be 10.5% according to the International Diabetes Federation Diabetes Atlas in 2021, with an escalating predicted epidemic trend of 12.2% and increased healthcare expenditure in 2045 (Sun et al., 2022). Understanding the pathophysiology and determining risk factors for diabetes have made great advances (Zheng et al., 2018). The gut microbiota has recently become a hot topic in metabolic disorders (Gurung et al., 2020). Although the clear microbial characteristics of diabetes have not been defined, once the composition of the gut microbiota is destroyed, an imbalanced gut microbial community may result in an abnormal production of metabolites, energy expenditure disturbance, insulin resistance (IR) and a low-grade chronic inflammatory state in diabetes (Qin et al., 2012; Gurung et al., 2020).

Metformin is the most prescribed oral antidiabetic agent for individuals with type 2 diabetes (T2D) due to its relative safety, low cost, and multiple beneficial effects on blood glucose, insulin sensitivity, lipid metabolism, cardiovascular mortality and tumorigenesis (Wu et al., 2017). However, its specific therapeutic targets that produce these complex beneficial effects are unknown. A large number of studies revealed that metformin treatment stimulated rapid changes in the composition of the gut microbiota, such as *Akkermansia* spp. (Wu et al., 2017; Ahmadi et al., 2020; Mueller et al., 2021). Metformin intervention beneficially altered gut microbial functions and host metabolic health, including improving short chain fatty acids (SCFAs) production, shaping of the bile acid pool, reducing lipopolysaccharide biosynthesis, and decreasing leaky gut and inflammation (Wu et al., 2017; Ahmadi et al., 2020; Mueller et al., 2021). Notably, the drug level of metformin in the gastrointestinal lumen is 30–300 times higher than the circulation levels, which indicates the complex molecular mechanism of metformin in the small intestine (McCreight et al., 2016).

Pioglitazone is another highly effective antidiabetic agent for T2D that is widely used as an insulin sensitizer and acts by activating peroxisome proliferator-activated receptor gamma, leading to the activation of various pathways associated with glycemic homeostasis and lipid metabolism (Ahmadian et al., 2013). A few experimental animal studies revealed that pioglitazone treatment slightly modified gut flora homeostasis (Bai et al., 2016; Li et al., 2019; Aggarwal et al., 2021). Pioglitazone reversed the high abundance of Proteobacteria in high-fat diet (HFD)-induced obese rats (Bai et al., 2016). Pioglitazone improved bacterial composition and structure and relieved intestinal inflammation and epithelial barrier impairment in a high fructose-fed mouse model (Li et al., 2019). Another experimental study revealed that pioglitazone intervention decreased microbial metabolites, such as hippurate and indole-3-ethanol, in iNOS knockout mice (Aggarwal et al., 2021). However, no clinical studies or experimental research explored pioglitazone-induced alterations in gut microbiota and fecal metabolites simultaneously. More evidence and the underlying mechanism await further investigation.

Due to the progressive nature of T2D, glycemic control with monotherapy is difficult to achieve for most diabetes patients, and combination therapy becomes essential (Deeks and Scott, 2006; Pharmacologic Approaches to Glycemic Treatment, 2021). A fixed-dose pioglitazone/metformin tablet has already been approved by United States and Europe for the treatment of T2D patients who have inadequate maintenance of glucose homeostasis with pioglitazone or metformin monotherapy or who receive a combination of pioglitazone and metformin (Deeks and Scott, 2006). Pioglitazone plus metformin demonstrated superior glycemic and lipid control efficacy than metformin plus placebo in a well-designed clinical trial (Deeks and Scott, 2006). However, the underlying mechanism of combination therapy is still unclear. According to previous evidence, we hypothesized that the combination of metformin and pioglitazone would play a synergetic role in the improvement of glucose and lipid metabolism *via* modifying gut microbiota and metabolites in HFD-induced obesity and IR mice. The present study evaluated the effects of metformin and pioglitazone combination therapy on gut microbiota and metabolites in HFD-fed mice.

## 2 Materials and methods

### 2.1 Animals and study design

C57BL/6 mice, 7-week-old males, were purchased from Beijing Vital River Laboratory Animal Technology Co., Ltd. (Beijing, China, SCXK-2021–0011). Mice were maintained in a standard specific pathogen-free environment (22 ± 2°C with a 12:12 h light:dark cycle) with free access to food and water. After adapting for 1 week, the mice were randomly categorized into two groups and fed for 10 weeks as follows: the control diet group (Ctr, *n*=6), which was raised on a normal control diet (AIN-93G) (10% of the calories as fat); and the HFD group (*n* = 25), which was raised on a HFD (D12492) (60% of the calories as fat). HFD group mice were randomly categorized into four groups with different pioglitazone or metformin interventions for 8 weeks: HFD control group (HFD, n = 7), which was continued on the HFD and received vehicle (0.5% carboxymethyl cellulose sodium); HFD with metformin intervention group (HFD+Met, n=6), which was continued on the HFD and received metformin (150 mg/kg/day body weight); HFD with pioglitazone intervention group (HFD+Pio, n=6), which was continued on the HFD and received pioglitazone (5 mg/kg/day body weight); and HFD with metformin and pioglitazone combination intervention group (HFD+Met+Pio, n=6), which was continued on the high-fat diet and treated with metformin (150 mg/kg/day body weight) combined with pioglitazone (5 mg/kg/day body weight). The Ctr group was pair-fed with the normal control diet and received vehicle for 8 weeks. Metformin (Hangzhou Zhongmei Huadong Pharmaceutical Co. Ltd., China) and pioglitazone (Hangzhou Zhongmei Huadong Pharmaceutical Co. Ltd., China) were dissolved with 0.5% carboxymethyl cellulose sodium separately and administered by intraperitoneal injection. The daily doses of 150 mg/kg metformin and 5 mg/kg pioglitazone we used were parallel to the doses used for combination therapy in humans in clinical studies and many previous studies (Haraguchi et al., 2008; Wang et al., 2016). At the end of the experimental period, all mice were sacrificed. Serum samples and cecal contents were collected for further analyses. The animal care and use committee of the Peking Union Medical College Hospital approved all of the procedures (Beijing, China, XHDW-2022–028). All of the animal surgeries were performed in compliance with the National Institutes of Health Guide for the Care and Use of Laboratory Animals.

### 2.2 Glucose and insulin tolerance tests

For the glucose tolerance test, mice fasted for 14 h were orally administered a glucose load (2 g/kg of body weight). For the insulin tolerance test, mice fasted for 6 h were injected intraperitoneally with short-acting insulin (1.0 U/kg of bodyweight). Blood glucose levels were monitored from tail bleeding before intervention and 15, 30, 60, 90, or further 120 min after intervention using a glucometer (FreeStyle Optium™, Abbott, United States). The area under the curve (AUC) was calculated as previously described (Liu et al., 2022a).

### 2.3 Serum biochemical parameter measurements

Blood samples were collected from the intraorbital retrobulbar plexus from mice after 14 h of fasting. The plasma was separated by centrifugation at 3,000 × g for 10 min at 4°C and stored at −80°C. An ELISA kit (ELR-Insulin-1, RayBiotech Life, Inc., Atlanta, United States) was used to detect plasma insulin levels. Plasma glucose, total triglyceride (TG), total cholesterol (TC) and high-density lipoprotein cholesterol (HDL-C) were measured using an autoanalyzer in Peking Union Medical College Hospital, as previously described (Liu et al., 2022a). Insulin sensitivity was analyzed using the homeostasis model assessment of insulin resistance (HOMA-IR). HOMA-IR was calculated as the fasting insulin concentration (μU/mL) × fasting glucose concentration (mmol/L)/22.5 (Matthews et al., 1985).

### 2.4 Gut microbiota 16S rDNA analysis

The DNA extraction and PCR amplification of the cecal content samples and the Illumina NovaSeq platform sequencing are described in our previous studies (Liu et al., 2022a). The 16S rDNA genes in distinct regions (16S V3-V4) were amplified using specific primers (341F, CCTAYGGGRBGCASCAG; 806R, GGACTACNNGGGTATCTAAT) and barcodes. For the effective tags obtained, denoising was performed using the deblur module in QIIME2 software (Version QIIME2-202006) to obtain initial amplicon sequence variants (ASVs) (Bokulich et al., 2013). Species annotation was performed using QIIME2 software. The absolute abundance of ASVs was normalized using a standard sequence number that corresponded to the sample with the fewest sequences. Subsequent analyses of alpha diversity and beta diversity were all performed based on the output normalized data. The alpha diversity indices, including the Shannon and Simpson indices, and beta diversity, including principal coordinate analysis (PCoA), among the five groups were calculated with QIIME2 and R software (Version 3.5.3). The discriminated bacterial taxa between groups were identified using linear discriminant analysis effect size (LEfSe) analysis (LDA score threshold: 3) (Segata et al., 2011).

### 2.5 Analysis of untargeted metabolomics

Liquid chromatography tandem mass spectrometry (LC-MS/MS) analyses were performed using an Ultra High Performance Liquid Chromatography (UHPLC) system (Vanquish, Thermo Fisher Scientific) with a UPLC BEH Amide column (2.1 mm × 100 mm, 1.7 μm) coupled to a Q Exactive HFX mass spectrometer (Orbitrap MS, Thermo) (Xue et al., 2021). The QE HFX mass spectrometer was used for its ability to acquire MS/MS spectra in information-dependent acquisition (IDA) mode in the control of the acquisition software (Xcalibur, Thermo). Metabolites were detected and analyzed in both positive- and negative-ion conditions to obtain the total ion current of the metabolites. After raw data were filtered and denoised, the final data set containing the peak number, sample name and normalized peak area information was imported into the SIMCA16.0.2 software package (Sartorius Stedim Data Analytics AB, Umea, Sweden) for multivariate analysis. Data were scaled and logarithmically transformed to minimize the impact of noise and high variance of the variables. Principal component analysis (PCA) was performed to visualize the distribution and grouping of the samples. KEGG (Kyoto Encyclopedia of Genes and Genomes) enrichment analysis was performed in Metaboanalyst 5.0.

### 2.6 Statistical analysis

All data are presented as the means ± standard deviation (SD). Statistics were analyzed using one-way analysis of variance (ANOVA) with Tukey’s post hoc analysis. A *p* value < 0.05 was considered statistically significant. Correlation analyses were performed using Spearman’s correlation coefficient test. Prism version 8.0 (GraphPad Software Inc., San Diego, CA, United States) was used for statistical analysis.

## 3 Results

### 3.1 Combination therapy with metformin and pioglitazone improves glucose and lipid metabolism in HFD-fed mice

**FIGURE 1:**
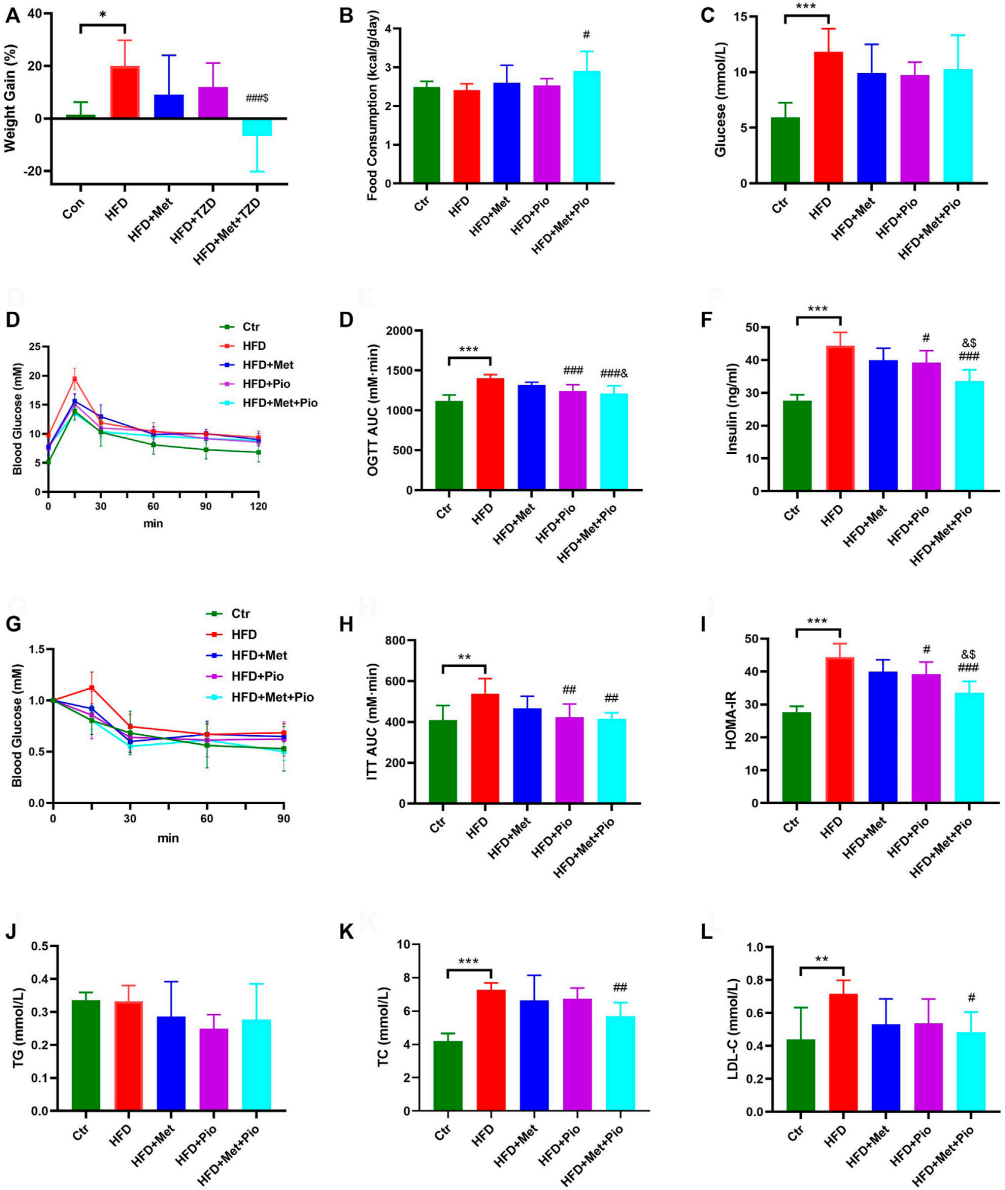
Glucose and lipid metabolism improvement with combination therapy of metformin and pioglitazone. **(A)** Weight Gain; **(B)** Food consumption; **(C)** Fasting serum glucose; **(D,E)** Oral glucose tolerance test and the area under the curve; **(F)** Fasting serum insulin; **(G,H)** Intraperitoneal insulin tolerance test and the area under the curve; **(I)** HOMA-IR; **(J)** Fasting serum TG; **(K)** Fasting serum TC; **(L)** Fasting serum LDL-C. Ctr, standard control diet; HFD, high-fat diet; HFD+Met, high-fat diet treated with metformin; HFD+Pio, high-fat diet treated with pioglitazone; HFD+Met+Pio, high-fat diet treated with metformin and pioglitazone. HOMA-IR, homeostasis model assessment of insulin resistance; TG, triglyceride; TC, total cholesterol; LDL-C, low-density lipoprotein cholesterol. Data are expressed as the means±SDs. (*n* = 6–7/group). One-way ANOVA; ^*^ *p* < 0.05, ^**^ *p* < 0.01, ^***^ *p* < 0.001 HFD *versus* Ctr group; ^#^ *p* < 0.05, ^##^ *p* < 0.01, ^###^ *p* < 0.001 HFD+Met group *versus* HFD group, HFD+Pio group *versus* HFD group, or HFD+Met+Pio group *versus* HFD group; ^&^ *p* < 0.05, ^and&^ *p* < 0.01, ^andand&^ *p* < 0.001 HFD+Pio group *versus* HFD+Met group, or HFD+Met+Pio group *versus* HFD+Met group; ^$^ *p* < 0.05, ^$$^ *p* < 0.01, ^$$$^ *p* < 0.001 HFD+Met+Pio group *versus* HFD+Pio group.

The mice were divided into five groups based on diet and pioglitazone or metformin intervention. There was no initial average weight difference between the five groups. At the end of the experimental period, the HFD group exhibited a significant increasing trend in body weight without an increase in food consumption (Figures 1A,B). After 8 weeks of treatment, the body weight gain percentage in the HFD+Met group and HFD+Pio group were lower compared to the HFD group. The HFD+Met+Pio combination therapy group demonstrated a significant body weight declines and increased food consumption. Except for the Ctr group, all of the other groups exhibited significantly increased fasting blood glucose levels (Figure 1C). Fasting glucose levels decreased in metformin and pioglitazone intervention groups were not significant. Metformin and pioglitazone intervention alleviated the impaired glucose tolerance in the HFD group as measured by the glucose tolerance test compared to the Ctr group (Figures 1D,E). Consistent with the results of glucose tolerance, fasting serum insulin levels, insulin tolerance tests and HOMA-IR of mice in the three treatment groups were significantly reduced compared to the HFD group (*p* < 0.05, Figures 1F–I). Notably, the HFD+Met+Pio group showed a more significant reduction in serum insulin levels and relieved HOMA-IR more effectively than the monotherapy groups, which evidently improved the insulin sensitivity (all *Ps* < 0.05). Notably, given that the animal models in our study were HFD-fed mice, mice might demonstrate more significant improvement in insulin resistance and glucose challenge test than fasting glucose. We further analyzed the effects of combination therapy on lipid metabolism (Figure 1J–L). TC and LDL-C levels were significantly increased in the HFD group and reduced in the metformin and pioglitazone combination therapy group. However, no significant differences in TG levels were detected between the five groups.

### 3.2 Monotherapy therapy with metformin and pioglitazone alters the gut microbiota in HFD-fed mice

To examine whether the 8-week intervention of metformin and pioglitazone affected the gut microbiota in HFD-fed mice, we assessed bacterial composition in cecal content samples using 16S rDNA amplicon sequencing-based analysis. Based on rarefaction curve analysis, the curves plateaued with increased sequence number, which suggested that the primary information of microbial species was ultimately obtained (Supplementary Figure S1A). Prior to investigating the detailed effects of combination therapy on gut microbiota composition, we first analyzed the microbial alterations of metformin and pioglitazone monotherapy. Metformin and pioglitazone monotherapy did not alter alpha diversity in HFD-fed mice according to the Simpson index and Shannon index (Supplementary Figure S1B). PCoA was performed to evaluate the beta diversity among the monotherapy groups. As shown in Supplementary Figure S1C-D, a clear separation between the Ctr and HFD-fed mice was presented. Metformin significantly altered gut microbiome composition profiles, and the effects of pioglitazone were relatively weak.

Of the top 10 phyla in metformin monotherapy shown in the community bar plot analysis (Supplementary Figure S2A), the three most abundant phyla were Verrucomicrobiota, Firmicutes and Bacteroidota. The heatmap of the top 30 genera in metformin monotherapy was shown in Supplementary Figure S2B. Metformin significantly increased the relative abundance of *Akkermansia,* Lachnospiraceae *NK4A136 group,* Prevotellaceae *UCG-001, Mucispirillum* and *UBA1819* but reduced the relative abundance of *Colidextribacter, Oscillibacter* and *Eubacterium_xylanophilum_group* (Supplementary Figure S2C-E). However, metformin did not reverse all of the microbes decreased in HFD, such as *Allobaculum* and *Faecalibaculum* (Supplementary Figure S2F). We further discriminated the individual bacterial taxa differentially enriched among the three groups identified with LEfSe analysis in Supplementary Figure S2G. At the species level, metformin treatment significantly harbored Lachnospiraceae *bacterium 28–4*, which has been found significantly changed in HFD mice when they were supplemented with tyrosol, one of the main polyphenolic compounds in extra virgin olive oil (Li et al., 2022)*.* Similar to metformin, the top 10 phyla in pioglitazone were shown in Supplementary Figure S3A. Firmicutes and Bacteroidota were the two most abundant phyla. The heatmap of the top 30 genera in pioglitazone monotherapy was shown in Supplementary Figure S3B. Pioglitazone significantly increased the relative abundance of microbes, including *Candidatus_Soleaferrea, Rikenella* and *UBA 1819* (Supplementary Figure S3C), and it reduced the relative abundance of several microbiota, such as Lachnospiraceae*_UCG-006* and *Oscillibacter* (Supplementary Figure S3D-E). Notably, pioglitazone failed to enhance the relative abundance of *Akkermansia* (Supplementary Figure S3F)*,* which was particularly increased in the metformin-treated group. LEfSe analysis of pioglitazone was shown in Supplementary Figure S3G. In addition to Lachnospiraceae *bacterium 28–4*, the pioglitazone group also harbored members of the species, including *Lactococcus lactis* and Ruminococcaceae *bacterium GD6*, which serves as the lactic acid producing bacteria (Kleerebezem et al., 2020).

### 3.3 Combination therapy with metformin and pioglitazone alters the gut microbiota in HFD-fed mice

**FIGURE 2:**
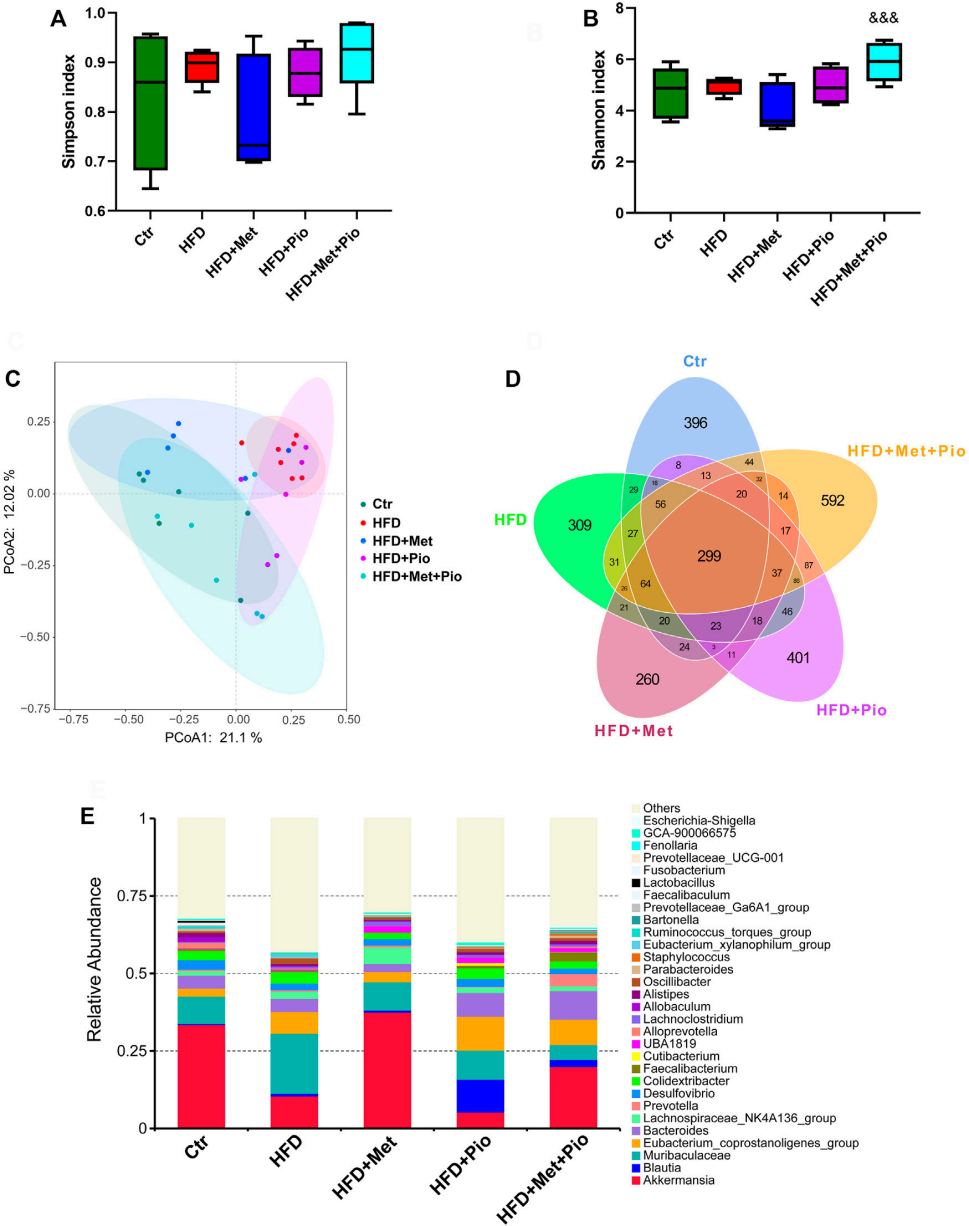
Combination therapy of metformin and pioglitazone on gut microbiota diversity. **(A)** Shannon index; **(B)** Simpson index; **(C)** PCoA plots of gut microbiome; **(D)** Venn diagram of the ASVs; **(E)** Relative abundance of the top 30 species at the genus level. Ctr, standard control diet; HFD, high-fat diet; HFD+Met, high-fat diet treated with metformin; HFD+Pio, high-fat diet treated with pioglitazone; HFD+Met+Pio, high-fat diet treated with metformin and pioglitazone. Data are expressed as the means±SDs. (*n* = 6–7/group). One-way ANOVA; ^*^ *p* < 0.05, ^**^ *p* < 0.01, ^***^ *p* < 0.001 HFD *versus* Ctr group; ^#^ *p* < 0.05, ^##^ *p* < 0.01, ^###^ *p* < 0.001 HFD+Met group *versus* HFD group, HFD+Pio group *versus* HFD group, or HFD+Met+Pio group *versus* HFD group; ^&^ *p* < 0.05, ^and&^ *p* < 0.01, ^andand&^ *p* < 0.001 HFD+Pio group *versus* HFD+Met group, or HFD+Met+Pio group *versus* HFD+Met group; ^$^ *p* < 0.05, ^$$^ *p* < 0.01, ^$$$^ *p* < 0.001 HFD+Met+Pio group *versus* HFD+Pio group.

As shown in Figures 2A,B, metformin and pioglitazone combination intervention significantly increased the alpha diversity of the gut microbiota according to the Simpson index. Notably, as shown in Figure 2C, metformin and pioglitazone combination intervention significantly modified the bacterial profiles in HFD-fed mice, and the HFD+Pio+Met group tended to be closer to the Ctr group than the other three groups, which indicated significant effects of the combination therapy on shaping intestinal microbial communities. A Venn diagram was used to visualize the number of shared and unique ASVs between the five groups (Figure 2D). The shared number of ASVs in all groups was 299, and the number of shared bacterial genera in the three treatment groups was 373. Venn diagrams revealed more unique ASVs in the HFD+Pio+Met group than the metformin or pioglitazone monotherapy groups. The three most abundant phyla enriched in the combination therapy group were Firmicutes, Bacteroidota and Verrucomicrobiota (Supplementary Figure S4A). The top 30 genera in all groups were shown in Figure 2F.

**FIGURE 3:**
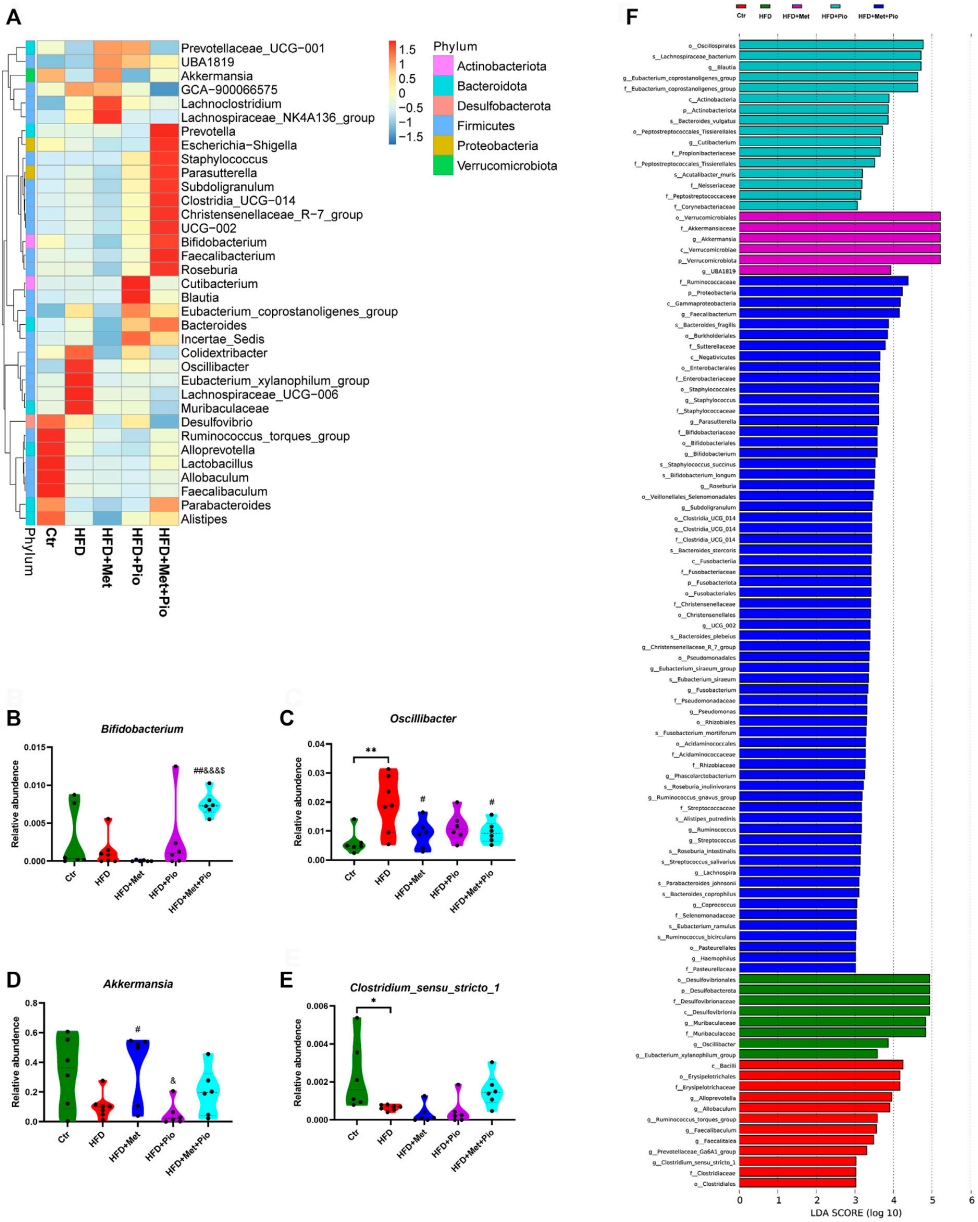
Changes in gut microbiota with combination therapy of metformin and pioglitazone. **(A)** Heatmap analysis of the different species at the genus level; **(B)** Relative abundance of *Bifidobacterium*; **(C)** Relative abundance of *Oscillibacter*; **(D)** Relative abundance of *Akkermansia*; **(E)** Relative abundance of *Clostridium_sensu_stricto_1*; **(F)** LEfSe analysis of the significantly enriched gut microbiome from the phylum level to the genus level. Ctr, standard control diet; HFD, high-fat diet; HFD+Met, high-fat diet treated with metformin; HFD+Pio, high-fat diet treated with pioglitazone; HFD+Met+Pio, high-fat diet treated with metformin and pioglitazone. Data are expressed as the means±SDs. (*n* = 6–7/group). One-way ANOVA; ^*^ *p* < 0.05, ^**^ *p* < 0.01, ^***^ *p* < 0.001 HFD *versus* Ctr group; ^#^ *p* < 0.05, ^##^ *p* < 0.01, ^###^ *p* < 0.001 HFD+Met group *versus* HFD group, HFD+Pio group *versus* HFD group, or HFD+Met+Pio group *versus* HFD group; ^&^ *p* < 0.05, ^and&^ *p* < 0.01, ^andand&^ *p* < 0.001 HFD+Pio group *versus* HFD+Met group, or HFD+Met+Pio group *versus* HFD+Met group; ^$^ *p* < 0.05, ^$$^ *p* < 0.01, ^$$$^ *p* < 0.001 HFD+Met+Pio group *versus* HFD+Pio group.

Then, heatmap of the top 30 genera showed that combination therapy significantly altered the relative abundance of a set of microbes (Figure 3A). Combination therapy intervention significantly increased the relative abundance of *Bifidobacterium,* Christensenellaceae*_R-7_group, Faecalibacterium* and *Roseburia* compared to the other four groups (Figure 3B, Supplementary Figure S4,S3B-E, all *Ps* < 0.05). In contrast, the relative abundances of *Oscillibacter, Eubacterium_xylanophilum_group* and Muribaculaceae were significantly reduced in the monotherapy and combination therapy groups (Figure 3C, Supplementary Figure S4,S3F-H). *Akkermansia* was significantly increased in the metformin monotherapy group and decreased in pioglitazone monotherapy, but showed only a mild change in the combination-treated group (Figure 3D). However still microbes could not be reversed by neither monotherapy nor combination therapy, such as Coriobacteriaceae*_UCG-002* and *Ruminococcus_torques_group*. LefSe analysis also identified a vast majority of differentially enriched bacteria in combination therapy mice.

### 3.4 Alteration of fecal metabolites in combination-treated mice

**FIGURE 4:**
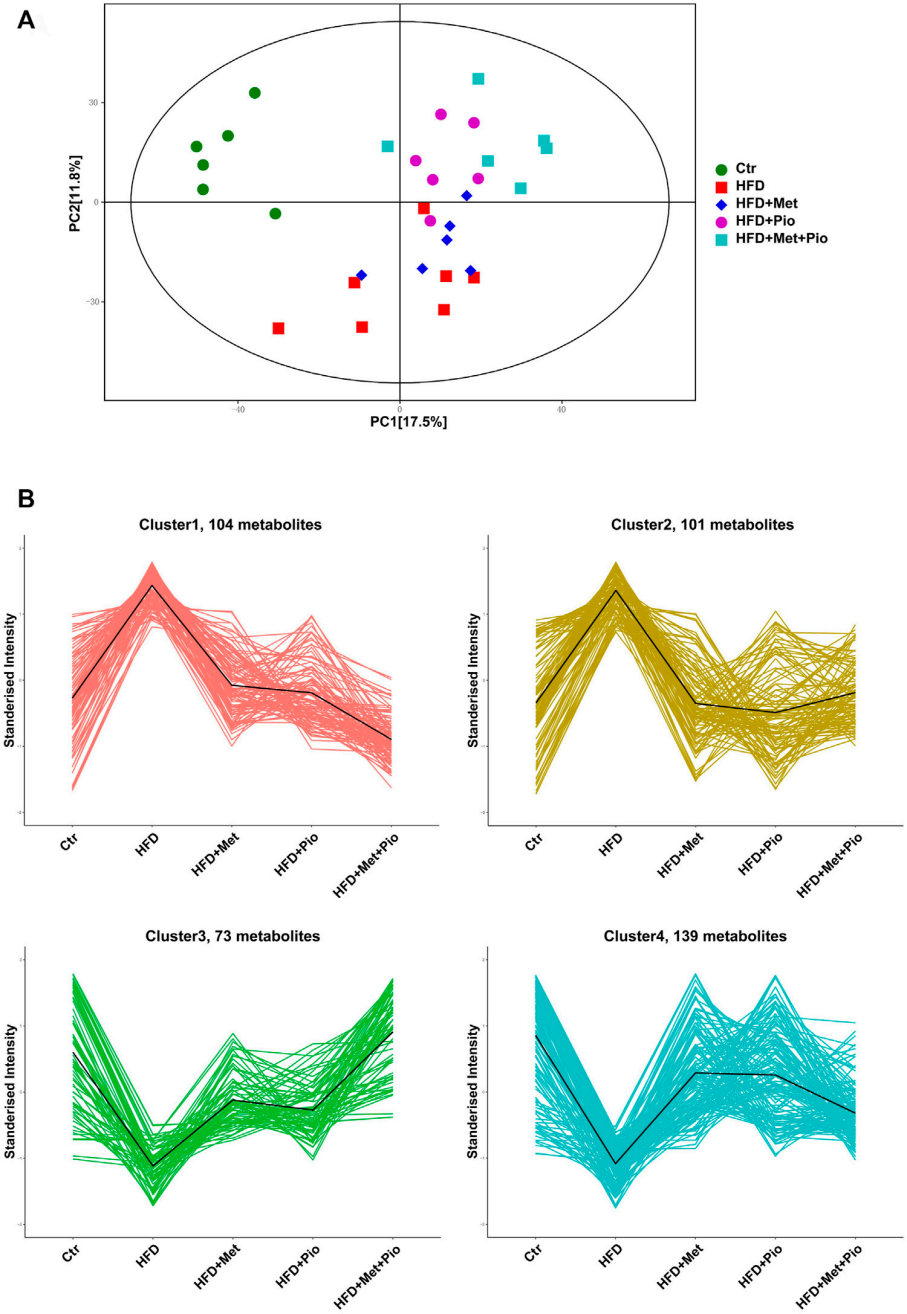
Cluster analysis of the gut metabolites. **(A)** PCA score plot; **(B)** Four clusters of the gut metabolites altered with combination therapy of metformin and pioglitazone by manual classification. Ctr, standard control diet; HFD, high-fat diet; HFD+Met, high-fat diet treated with metformin; HFD+Pio, high-fat diet treated with pioglitazone; HFD+Met+Pio, high-fat diet treated with metformin and pioglitazone.

Multiple metabolites of gut flora interact with host as functional signaling molecules affecting host energy metabolism. Untargeted metabolomics analysis of the fecal samples was performed to explore possible alterations of fecal metabolites in the combination-treated group. A total of 1075 metabolites were identified, and a pie plot of metabolite classification and proportion was shown in Supplementary Figure S5A. Compared by PCA, the combination-treated group showed a clearer discrimination with HFD than metformin or pioglitazone monotherapy (Figure 4A). The score plot indicated an x-axis separating the Ctr group from the HFD, HFD+Met, HFD+Pio and HFD+Met+Pio groups. The HFD+Met+Pio group was rather separated along the y-axis. K-means cluster analysis using the Hartigan–Wong algorithm was performed to classify fecal metabolites (giving k = 6, Supplementary Figure S5B). To explore the variation trends of metabolites corresponding to gut microbiota alterations in detail, we manually clustered metabolites again (Figure 4B). Clusters 1 and 2 consisted of metabolites that were increased in the HFD group and reversed in the monotherapy and combination therapy groups. Metabolites in Cluster 1 exhibited a more distinct reverse trend in the combination-treated group than in the metformin and pioglitazone monotherapy groups. Similar to Clusters 1 and 2, Clusters 3 and 4 represented metabolites that were decreased in HFD and reversed by treatment. Metabolites in Cluster 3 exhibited a more distinct reversal trend in the combination therapy group.

**FIGURE 5:**
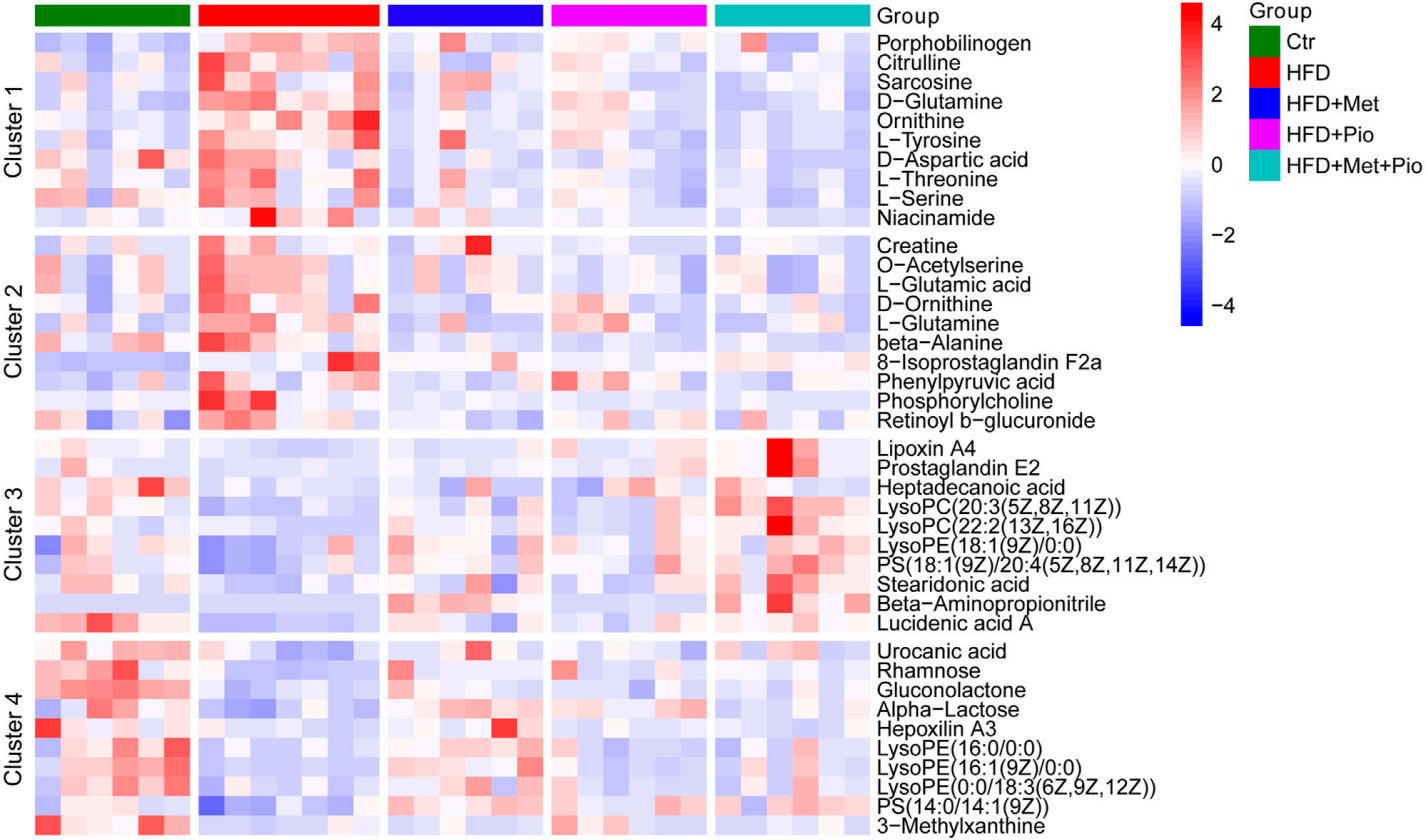
Heatmap analysis of the representative altered gut metabolites in Clusters 1–4. Ctr, standard control diet; HFD, high-fat diet; HFD+Met, high-fat diet treated with metformin; HFD+Pio, high-fat diet treated with pioglitazone; HFD+Met+Pio, high-fat diet treated with metformin and pioglitazone.

Based on ANOVA, metabolites significantly altered or with high relative concentrations in Clusters 1-4 were further analyzed using KEGG enrichment analysis respectively (Supplementary Figure S6). The metabolites in Clusters 1 and 2 were primarily involved in pathways associated with amino acid metabolism, such as D-amino acid metabolism, biosynthesis of amino acids, protein digestion and absorption, and alanine, aspartate and glutamate metabolism. The metabolites in Clusters 3 and 4 were involved in pathways associated with lipid metabolism, such as arachidonic acid metabolism and regulation of lipolysis in adipocytes. Corresponding with the cluster analysis in Figure 4B and KEGG enrichment analysis in Supplementary Figure S6, 10 metabolites from each cluster primarily based on KEGG enrichment data were shown in the heatmap (Figure 5).

### 3.5 Associations between altered gut microbiota and metabolites

**FIGURE 6:**
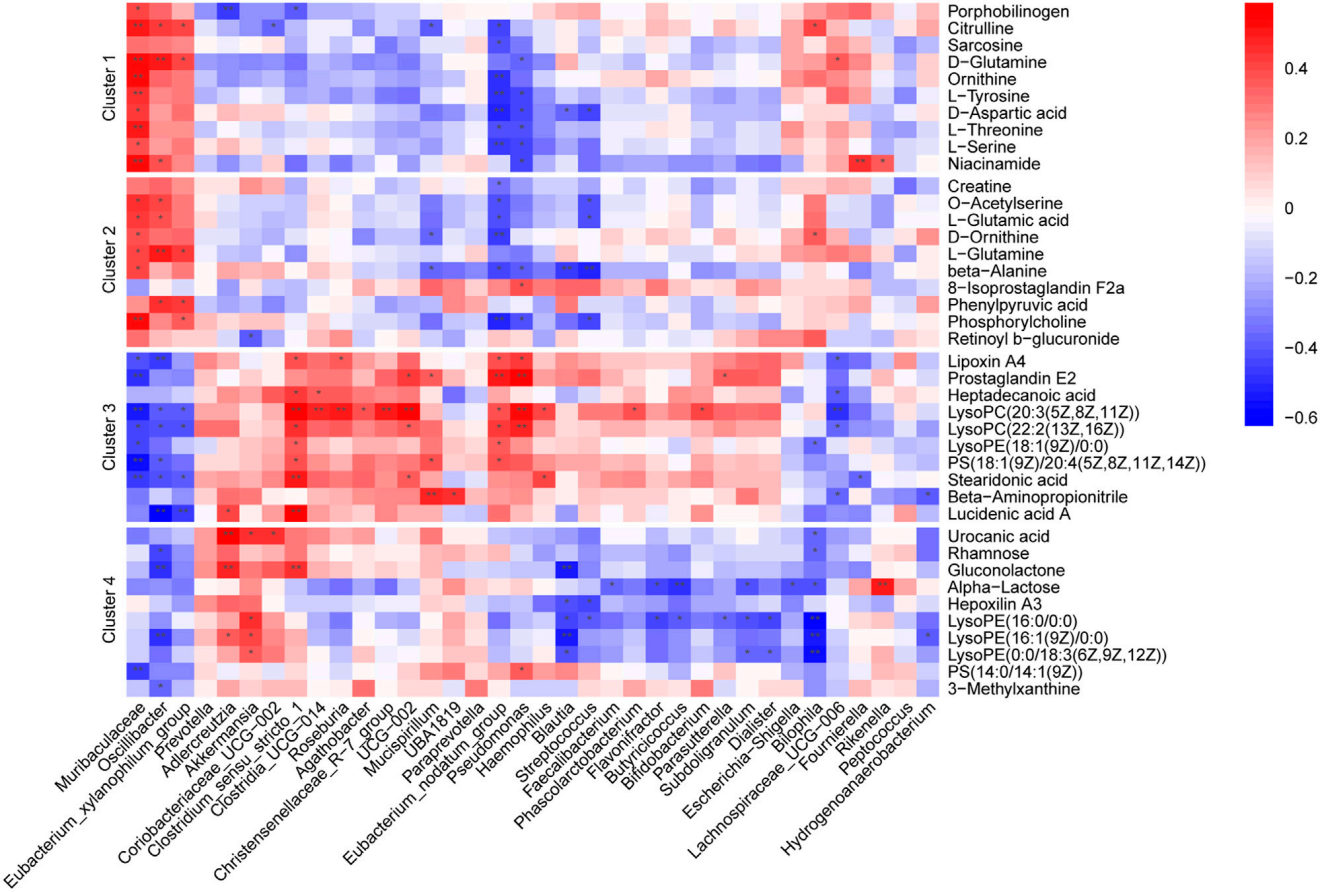
Heatmaps of Spearman correlation analysis between altered gut microbes and metabolites. Crosses show the correlation coefficients (r), ^*^ *p* < 0.05, ^**^ *p* < 0.01.

To explore the associations between altered gut microflora and metabolites, we performed a correlation analysis between the 40 selected metabolites and 36 significantly altered gut microbiota at the genus level (ANOVA *p* < 0.05) in all groups using Spearman analysis (Figure 6). Our analysis showed that the abundance alterations of Muribaculaceae*, Oscillibacte* and *Eubacterium_xylanophilum_group* positively correlated with metabolites in Clusters 1 and 2 but negatively correlated with metabolites in Clusters 3 and 4. In contrast, gut microbiota, including *Eubacterium_nodatum_group, Akkermansia, Clostridium_sensu_stricto_1* and *Roseburia,* negatively correlated with metabolites in Clusters 1 and 2 but positively correlated with metabolites in Clusters 3 and 4. The close relationship between the microbiota and fecal metabolites suggested that the alteration tendencies of metabolites may be at least indirectly mediated by corresponding gut microbes.

### 3.6 Associations between gut microbiota metabolites and glucolipid metabolic parameters

**FIGURE 7:**
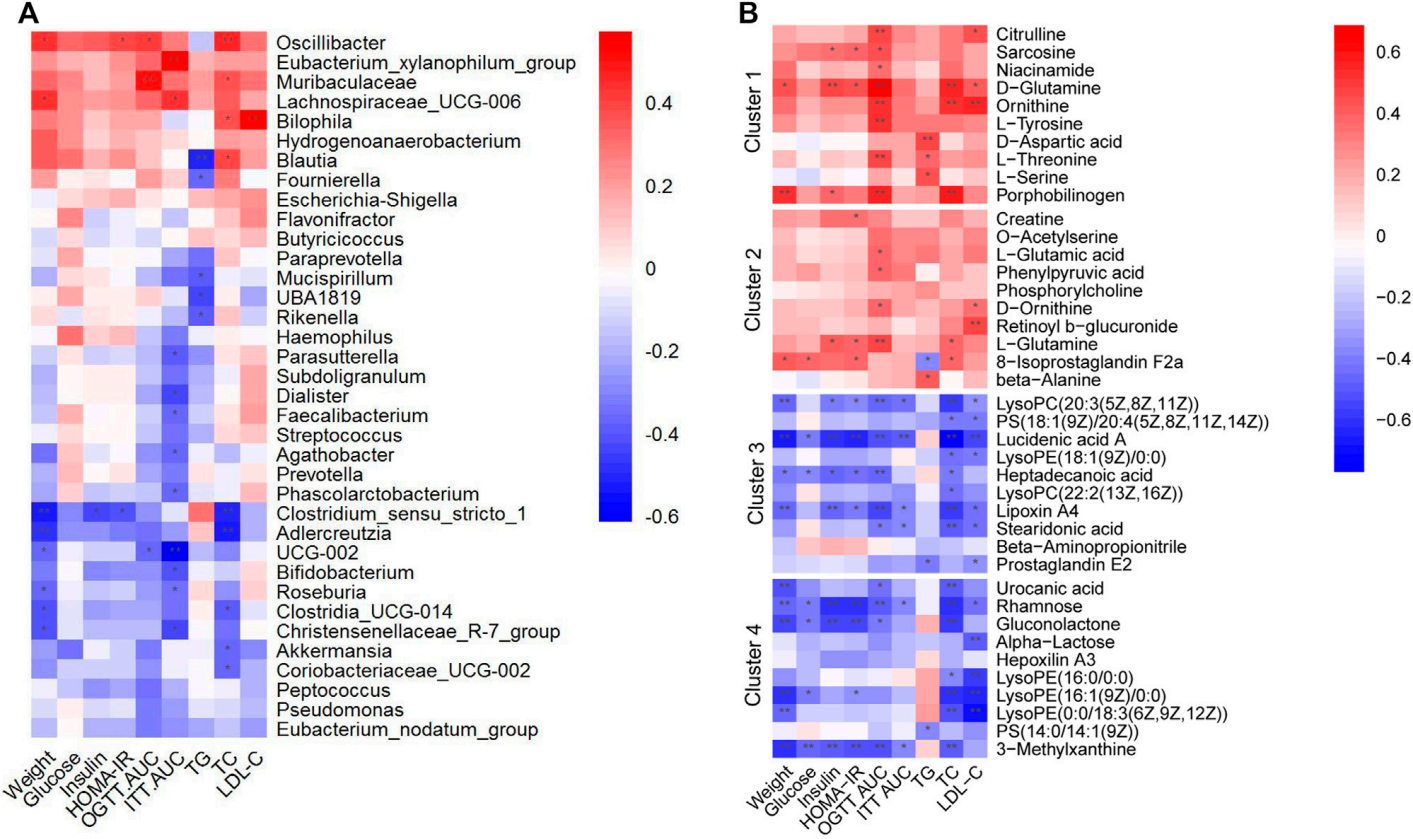
Heatmaps of Spearman correlation analysis between altered gut microbes, gut metabolites and glucolipid metabolic parameters. **(A)** Correlation analysis between altered gut microbes and glucolipid metabolic parameters. **(B)** Correlation analysis between altered gut metabolites and glucolipid metabolic parameters. Crosses show the correlation coefficients (r), ^*^ *p* < 0.05, ^**^ *p* < 0.01.

To further determine whether the alterations of gut microbiota and fecal metabolites were associated with glucolipid metabolism intervened with metformin and pioglitazone, the link between gut microbiota and fecal metabolites and glucolipid metabolic parameters in all groups were investigated. As shown in Figure 7A, a number of enriched gut microbiota in the combination-treated group, such as *Bifidobacterium,* Christensenellaceae*_R-7_group, Akkermansia, Eubacterium_nodatum_group,* and *Roseburia*, were negatively associated with glucolipid metabolic parameters. Decreased bacteria, such as *Oscillibacte, Eubacterium_xylanophilum_group* and *Bilophila,* were positively associated with glucolipid metabolic parameters. Correspondingly, as shown in Figure 7B, metabolites in Clusters 1 and 2 were positively associated with glucolipid metabolic parameters, and Clusters 3 and 4 were negatively associated with glucolipid metabolic parameters, which indicated that metabolic improvement in the combination of metformin and pioglitazone may be partially mediated by gut microbiota and fecal metabolites in Clusters 1-4.

## 4 Discussion

The gut microbiota has recently become a hot topic in metabolic disorders (Gurung et al., 2020). A substantial body of research have found that T2D is characterized by gut dysbiosis and disturbed intestinal metabolites (Gurung et al., 2020). Though the core gut microbial features of T2D vary regarding genetic inheritance, diet and lifestyle factors, the beneficial genera of *Bifidobacterium, Lactobacillus, Faecalibacterium, Roseburia* and *Akkermansia* are negatively associated with T2D, whereas the genera of *Blautia*, *Fusobacterium, Desulfovibrio,* and some species of *Bacteroides* are positively associated with T2D (Qin et al., 2012; Gurung et al., 2020). Previous studies demonstrated that metformin modified the gut microbiota and altered gastrointestinal and circulation metabolite profiles to further regulate metabolism (Wu et al., 2017; Ahmadi et al., 2020; Mueller et al., 2021). Several animal studies revealed that pioglitazone may slightly alter gut microbiota or metabolites to improve metabolic health (Bai et al., 2016; Li et al., 2019; Aggarwal et al., 2021). Consistent with previous studies, we found that the abundance of several beneficial microbes increased while conditional pathogens were reduced in the metformin and pioglitazone monotherapy groups. We found that the abundance of the beneficial microbe *Akkermansia* increased in the metformin monotherapy group, and the abundance of several harmful microbiota decreased, such as *Eubacterium_xylanophilum_group* and *Oscillibacter* (Qin et al., 2012; Kong et al., 2019; Gurung et al., 2020; Peng et al., 2022). *Akkermansia* increased, particularly during metformin treatment, in clinical and experimental studies and functioned as a SCFAs-producing and mucin-degrading bacterium (Ke et al., 2021). *Eubacterium_xylanophilum_group* was found to have increased abundance in ulcerative colitis mice (Peng et al., 2022). *Oscillibacter* was found a conditional pathogen positively correlated with obesity- and nutrient-related metabolic pathways in high-sucrose diet-fed mice and inflammatory cytokine levels in ulcerative colitis mice (Kong et al., 2019; Peng et al., 2022). Similar to the metformin monotherapy group, *Oscillibacter* was also reduced in the pioglitazone monotherapy group. Lachnospiraceae*_UCG-006* was also reduced in the pioglitazone monotherapy group. It was found highly potentially associated with diet-induced obesity and nonalcoholic fatty liver disease-related parameters in serum and liver in previous studies (Li et al., 2020; Duan et al., 2022). In addition, several beneficial microbes, such as *Candidatus_Soleaferrea*, were increased in pioglitazone monotherapy group (Zou et al., 2022). Briefly, both metformin and pioglitazone monotherapy revealed significant effects on gut microbiota modification in our HFD-fed mice. However, the combined effect of metformin and pioglitazone on gut microbiota has not been explored. Therefore, we further evaluated the combined effect on gut microbiota alterations in HFD-fed mice.

Consistent with better improved glucose and lipid control, including OGTT, ITT, HOMA-IR, TC and LDL-C, gut microbiota alterations were evidently improved in the combined treatment group. A set of well-documented beneficial microbes or prebiotics, including *Bifidobacterium, Faecalibacterium* and *Roseburia,* especially thrived in the combined treatment group (Kong et al., 2019; Gurung et al., 2020). *Bifidobacterium* was proved to be the most potentially protective genus as probiotics against T2D with sufficient evidence, including SCFAs-production, vitamin and cofactor production and gut microbiome regulation (Kong et al., 2019; Gurung et al., 2020). *Faecalibacterium* and *Roseburia* were well documented beneficial microbes for their SCFAs-producing signature (Gurung et al., 2020). Christensenellaceae*_R-7_group, Clostridia_UCG-014* and *Parasutterella* were all increased in the combined treatment group. Evidence showed that Christensenellaceae*_R-7_group* was increased with a special traditional Chinese medicine capsule intervention, which could ameliorated hyperglycemia and pathological changes in diabetic nephropathy in rat models (Su et al., 2021). *Clostridia_UCG-014* was identified as a probiotic associated with tryptophan metabolism in colitis mice and might mediate the improvement of gut barrier disruption, colon inflammation and the pathological phenotype (Yang et al., 2021). *Parasutterella* was identified as a core component of the gut microbiota in humans and mice and is associated with various health outcomes in bile acid profiles and cholesterol metabolism (Ju et al., 2019). Conversely, several harmful microbes were reduced, such as *Oscillibacter, Eubacterium_xylanophilum_group* and Lachnospiraceae*_UCG-006* (Kong et al., 2019; Li et al., 2020; Duan et al., 2022; Peng et al., 2022). Therefore, the alterations of these beneficial and harmful microbes indicate significant synergistic effects of combination therapy on gut microbiota modification. However, not all of the potential beneficial microbes that were decreased in HFD can be reversed by combined treatment, such as *Lactobacillus, Clostridium_sensu_stricto_1* and Coriobacteriaceae*_UCG-002* (Kong et al., 2019; Sultana et al., 2022). Notably, *Akkermansia,* as a beneficial microbiota, showed different trends in the metformin and pioglitazone monotherapy groups and was slightly increased in the combined treatment group, which suggests another form of complementary action between metformin and pioglitazone.

Gut microbiota interact with the host *via* the production of a diverse reservoir of metabolites (Agus et al., 2021). Increased beneficial microbes are vital for the production of beneficial metabolites, such as SCFAs, vitamins and cofactors, and reduced harmful microbes are accompanied by reduced glycolipid lipopolysaccharides or trimethylamine-N-oxide (Kong et al., 2019; Gurung et al., 2020; Agus et al., 2021). Therefore, we used untargeted metabolomics analysis of the fecal samples to explore the possible alterations of fecal metabolites in the combination-treated group. Metabolites that were significantly increased in the HFD group and reversed in the treatment group primarily consisted of amino acid metabolism, including metabolites that exhibited a more distinct reverse trend in the combination-treated group, such as citrulline, sarcosine, D-glutamine, D-aspartic acid and L-tyrosine. KEGG enrichment analysis of these metabolites primarily included amino acid metabolism pathways. A previous study showed that fecal amino acids demonstrated an increasing trend in HFD-fed mice (Zeng et al., 2020; Agus et al., 2021; Qiu et al., 2021). Correlations among altered microbes, amino acids and glucolipid metabolic parameters in our results hinted that altered gut microbiota might at least indirectly reduce intestinal amino acid levels to improve metabolic health in combination-treated group. However, the fecal metabolic profiles are dependent on multiple factors, including diet, gut bacteria composition, different gut segments, turnover of gut intestinal cells and absorption function (Agus et al., 2021; Cai et al., 2021; Qiu et al., 2021; Bethancourt et al., 2022). There are still not enough researches have explored whether the altered bacteria take up or produce specific amino acids (Agus et al., 2021). Previous studies found that increased serum amino acid levels were associated with IR in obesity and diabetes, but the underlying mechanisms are still unclear (Bloomgarden, 2018). Therefore, the potential pathogenic mechanism of increased amino acids in HFD mice needs further investigation.

On the contrary, metabolites that were significantly reduced in the HFD group and reversed in the treatment group primarily consisted of products of lipid metabolism, including metabolites that exhibited a more distinct reversal trend in the combination-treated group, such as lipoxin A4, prostaglandin E2, stearidonic acid, lucidenic acid A, heptadecanoic acid and LysoPCs. KEGG enrichment analysis of these metabolites primarily included lipids metabolic pathways, such as the arachidonic acid metabolism pathway. As a product of arachidonic acid metabolism, lipoxin A4 exhibits strong anti-inflammatory properties and protected against obesity-induced systemic disorders in previous studies (Börgeson et al., 2015; Shen et al., 2022). Lipoxin A4 intervention increased the abundance of beneficial bacteria and SCFAs-production and bile acids pool alterations in type 1 diabetes rats (Shen et al., 2022). Most likely due to the different experimental designs, the lipoxin A4-related flora in our results were different at the genus level from the previous type 1 diabetes model (Shen et al., 2022). As an omega-3 polyunsaturated fatty acid that is rich in fish oil, stearidonic acid suppressed lipid accumulation in adipocytes, prevented the development of T2DM and enhanced metabolic health in previous studies, which suggests its usage as a dietary supplement for obesity (Caesar et al., 2015; Li et al., 2017). Supplementation with fish oil modified the gut microbiome in a mouse model and increased the abundance of *Akkermansia muciniphila* (Caesar et al., 2015). Similar to lipoxin A4 and stearidonic acid, other metabolites, such as prostaglandin E2 and heptadecanoic acid, have been revealed beneficial metabolic effects in obesity (Bethancourt et al., 2022; Wang et al., 2022). Notably, glycerophospholipids, such as lysoPC(20:3 (5Z,8Z, 11Z)) and LysoPE (18:1 (9Z)/0:0), also exhibited increasing tendencies in the therapy groups. Alterations in glycerophospholipids and their correlations with gut microbiota in previous research are inconsistent (Xue et al., 2020; Liu et al., 2022b). Further research is needed to precisely clarify the associations between gut microbiota and glycerophospholipid metabolism in the pathogenesis of metabolic diseases.

Pearson’s correlation analysis showed that lipid metabolic metabolites increased in the combined treatment group and were positively associated with an increase in beneficial microbes, such as *Bifidobacterium,* Christensenellaceae*_R-7_group, Akkermansia, Eubacterium_nodatum_group,* and *Roseburia*, and negatively associated with glucolipid metabolic parameters. Metabolites that were decreased in the combined treatment group were inversely associated. These results suggested that altered gut microbiota at least indirectly reduced intestinal amino acid levels and increased lipid metabolism to improve metabolic health in the combination-treated group. The underlying mechanism and the extent to which the altered fecal metabolites were mediated by gut microbiota in our results requires further investigation.

Meanwhile, there are still some limitations in our present study. Firstly, the assessment of drug combinations requires a quantitative approach that begins with the individual dose-effect curves from which the combined additive effect is calculated. The fixed-dose pioglitazone/metformin tablet approved in the United States and Europe is 15mg/500 mg or 15mg/850 mg (Deeks and Scott, 2006). However, we only selected one of fixed-dose of pioglitazone and metformin (15mg/500 mg). The underling gut microbiota alteration of 15mg/850 mg pioglitazone/metformin tablet might be slightly different. Secondly, untargeted metabolomics analysis was executed to explore the possible alterations of fecal metabolites in the present study. Unfortunately, SCFAs, which are the major microbial metabolites produced by dietary fiber fermentation within the intestinal lumen and have been identified as vital mediators in maintaining intestinal immunity and systemic inflammation (Dalile et al., 2019), were not detected by LC-MS/MS analyses using an UHPLC system.

In summary, the data of our present study revealed a synergistic, or at least a beneficial additive, effect of the combination of metformin and pioglitazone on the improvement of glucose and lipid metabolism and insulin sensitivity in HFD-induced obesity and IR mice. The beneficial effect may be partially mediated by an increase in beneficial microbiota and a reduction in harmful microbiota, with altered fecal metabolite profiles, including increased lipid metabolism and decreased amino acid metabolism. However, to what extent the hypoglycemic effect of combination therapy comes from its modification of the gut microbiota is not clear, and the underlying crosstalk mechanisms of microbiota and fecal metabolites are also unclear. Diet and gut bacterial composition are vital sources of gut metabolites. Few studies have explored whether the altered bacteria take up or produce specific metabolites. Therefore, we expect future in-depth research on synergistic metabolic interactions between metformin and pioglitazone on gut microbiota and the remodeling of microbiota-derived metabolome.

## Data Availability

The datasets presented in this study can be found in online repositories. The names of the repository/repositories and accession number(s) can be found below: https://www.ncbi.nlm.nih.gov; SAMN30409775-SAMN30409805.
