# Supplementary material for: Potential benefits of metformin and pioglitazone combination therapy via gut microbiota and metabolites in high-fat diet-fed mice
Source: Front Pharmacol. 2022 Oct 11;13:1004617. doi: 10.3389/fphar.2022.1004617 (PMC9592694; doi:10.3389/fphar.2022.1004617)
Supplement: Supplementary file 1 [file DataSheet1.docx]

Supplementary Material

# Supplementary Figures

**
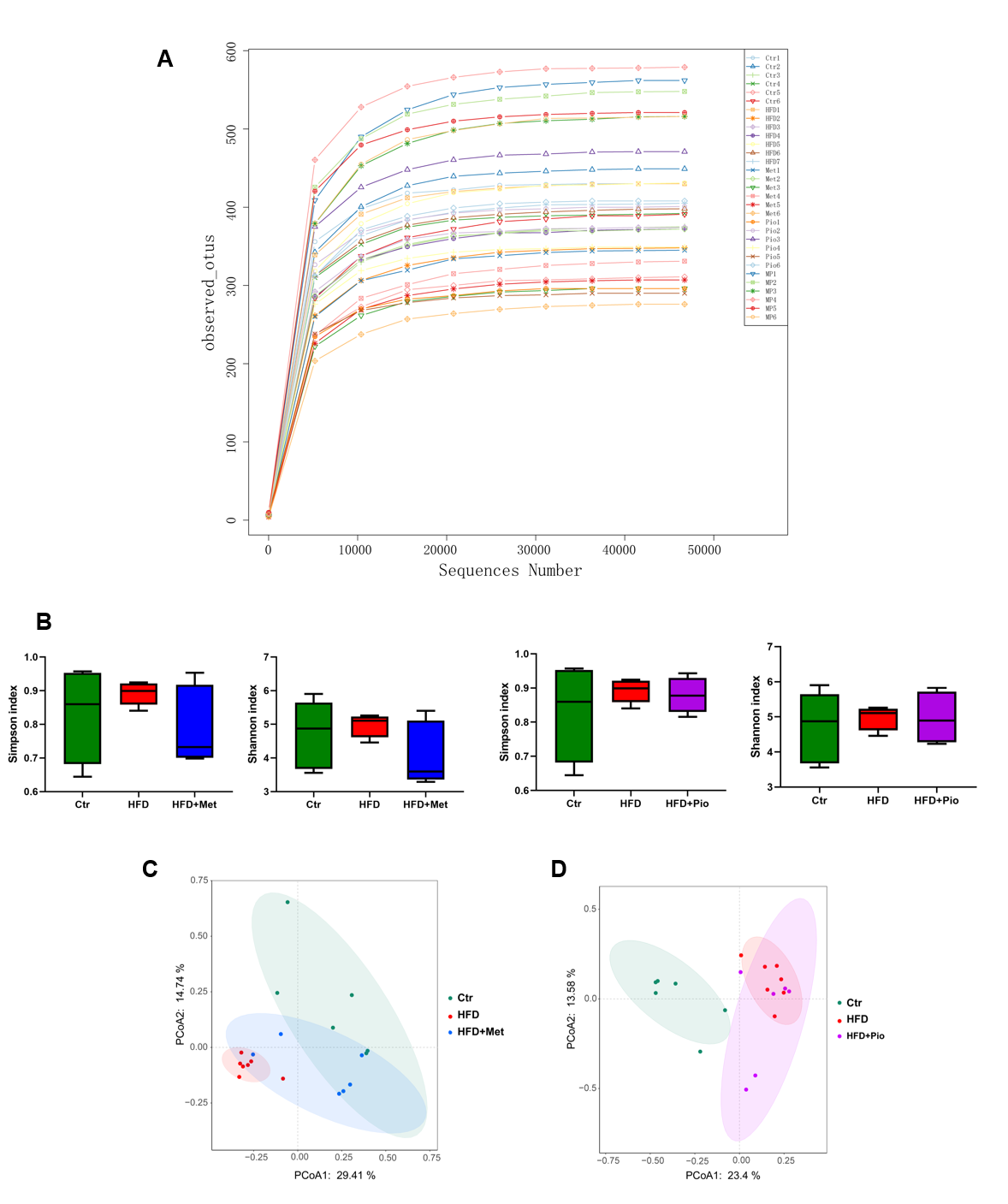
**

**Figure S1. Alpha diversity and beta diversity of gut microbiota alterations in monotherapy therapy.** (A) Rarefaction curve analysis; (B) Shannon index and Simpson index; (C) PCoA plots of the gut microbiome in metformin monotherapy; (D) PCoA plots of the gut microbiome in pioglitazone monotherapy. Ctr, standard control diet; HFD, high-fat diet; Met, high-fat diet treated with metformin; Pio, high-fat diet treated with pioglitazone; MP, high-fat diet treated with metformin and pioglitazone; HFD+Met, high-fat diet treated with metformin; HFD+Pio, high-fat diet treated with pioglitazone.


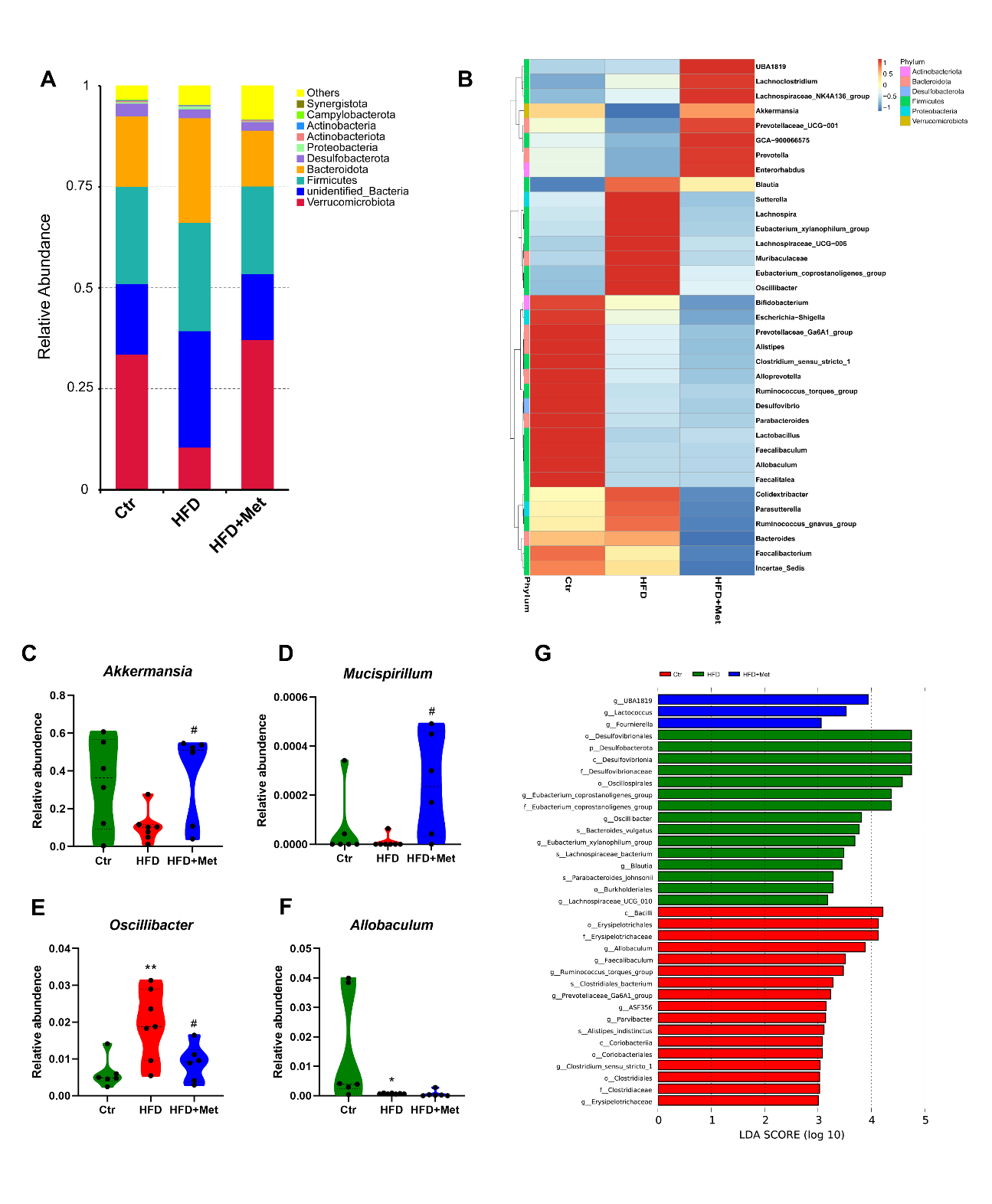


**Figure S2. Alteration of gut microbiota with metformin monotherapy.** (A) Relative abundance at the phylum level. (B) Heatmap analysis of the different species at the genus level; (C-F) Relative abundance of four altered microbiota; (G) LEfSe analysis of the significantly enriched gut microbiome from the phylum level to the genus level. Ctr, standard control diet; HFD, high-fat diet; HFD+Met, high-fat diet treated with metformin. Data are expressed as the means±SDs. (n = 6-7/group). One-way ANOVA; ^*^*P*< 0.05, ^**^*P* < 0.01, ^***^*P* < 0.001 HFD versus Ctr group; ^#^*P*< 0.05, ^##^*P* < 0.01, ^###^*P* < 0.001 HFD+Met group versus HFD group.


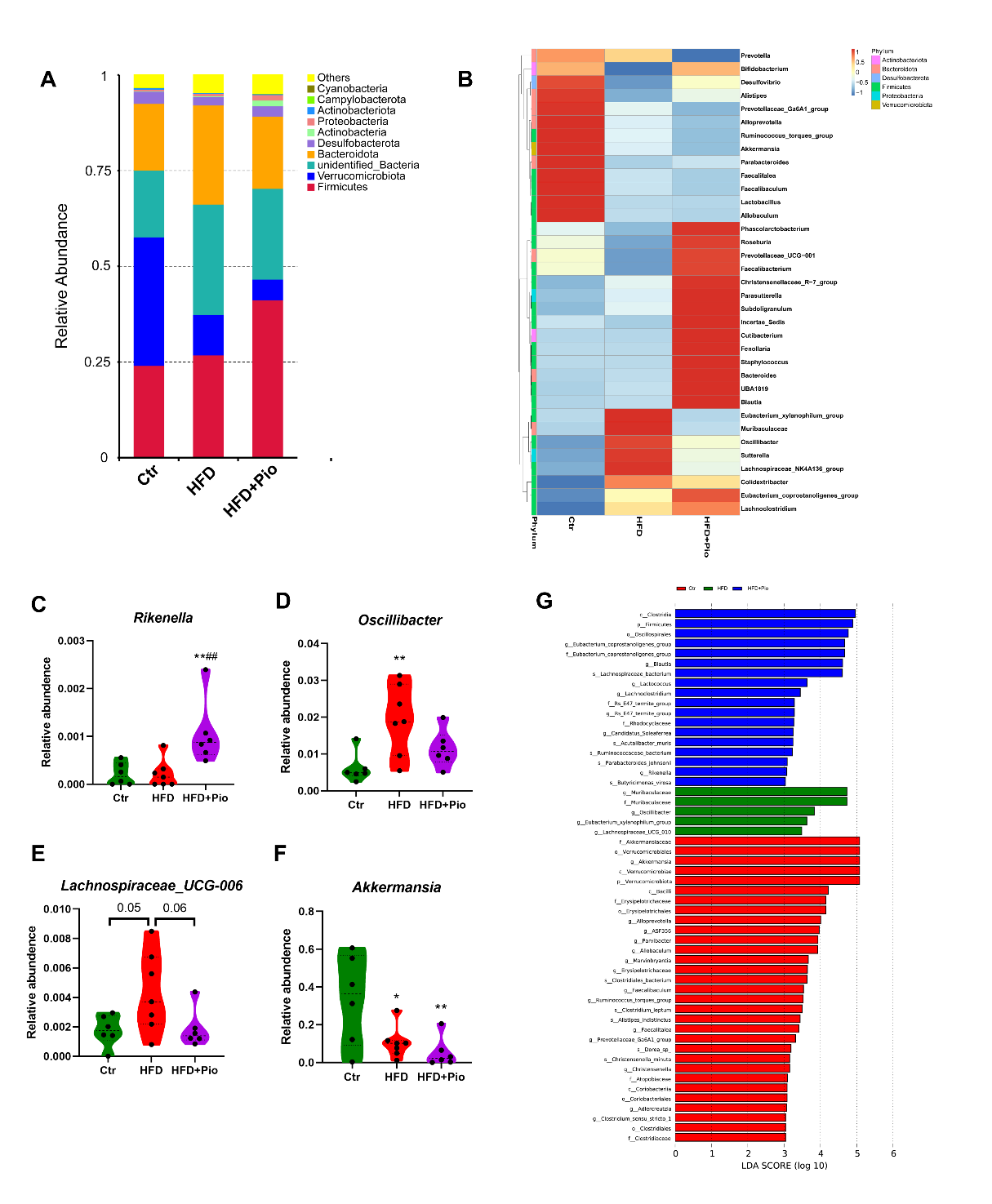


**Figure S3. Alteration of gut microbiota with pioglitazone monotherapy.** (A) Relative abundance at the phylum level. (B) Heatmap analysis of the different species at the genus level; (C-F) Relative abundance of four altered microbiota; (G) LEfSe analysis of the significantly enriched gut microbiome from the phylum level to the genus level. Ctr, standard control diet; HFD, high-fat diet; HFD+Pio, high-fat diet treated with pioglitazone. Data are expressed as the means±SDs. (n = 6-7/group). One-way ANOVA; ^*^*P*< 0.05, ^**^*P* < 0.01, ^***^*P* < 0.001 HFD versus Ctr group; ^#^*P*< 0.05, ^##^*P* < 0.01, ^###^*P* < 0.001 HFD+Pio group versus HFD group


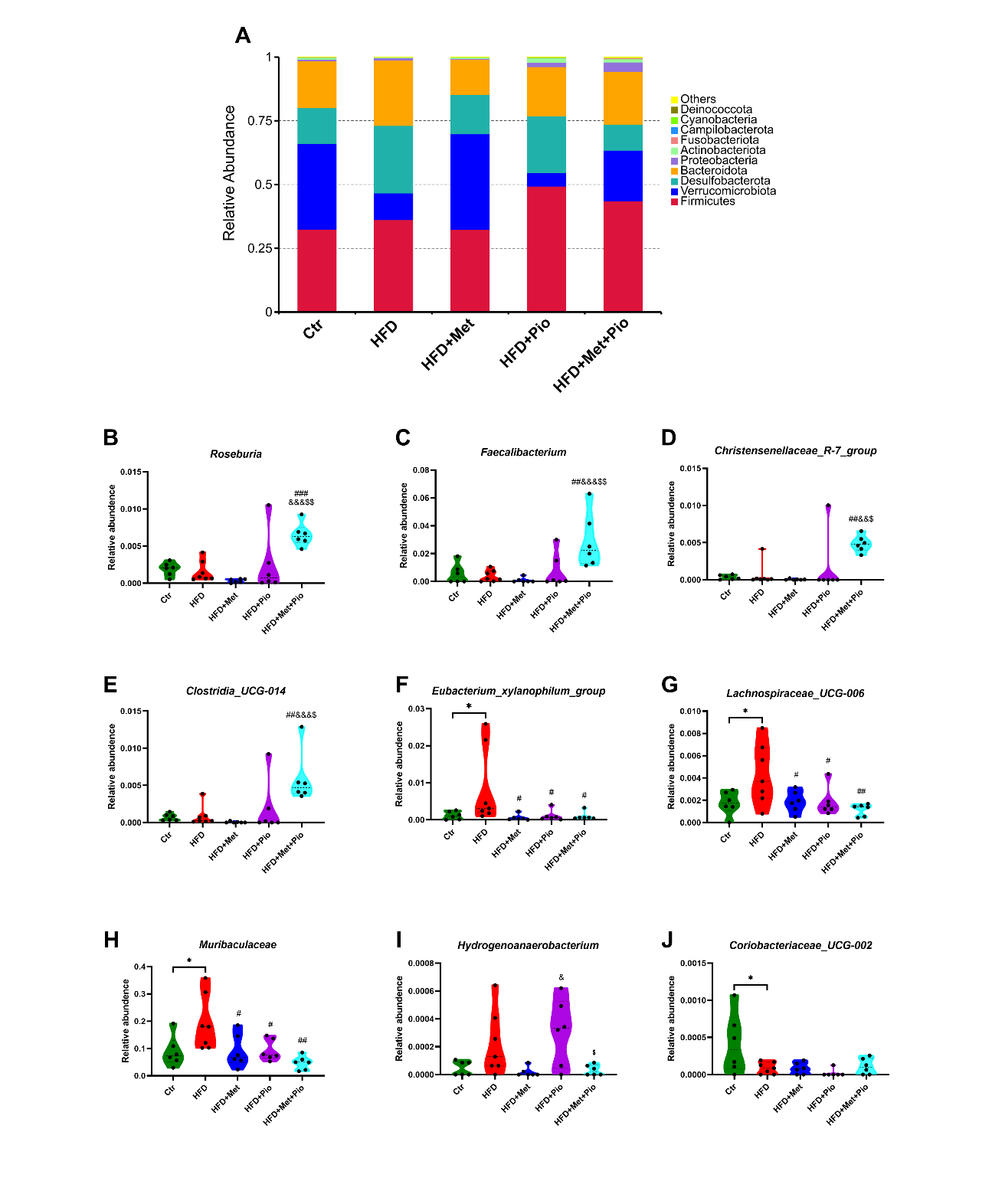


**Figure S4.** **The alteration of gut microbiota with combination therapy of metformin and pioglitazone.** (A) Relative abundance at the phylum level. (B-J) Relative abundance of several altered microbiota. Ctr, standard control diet; HFD, high-fat diet; HFD+Met, high-fat diet treated with metformin; HFD+Pio, high-fat diet treated with pioglitazone; HFD+Met+Pio, high-fat diet treated with metformin and pioglitazone. Data are expressed as the means±SDs. (n = 6-7/group). One-way ANOVA; ^*^*P*< 0.05, ^**^*P* < 0.01, ^***^*P* < 0.001 HFD versus Ctr group; ^#^*P*< 0.05, ^##^*P* < 0.01, ^###^*P* < 0.001 HFD+Met group versus HFD group, HFD+Pio group versus HFD group, or HFD+Met+Pio group versus HFD group; ^&^*P*< 0.05, ^&&^*P* < 0.01, ^&&&^*P* < 0.001 HFD+Pio group versus HFD+Met group, or HFD+Met+Pio group versus HFD+Met group; ^$^*P*< 0.05, ^$$^*P* < 0.01, ^$$$^*P* < 0.001 HFD+Met+Pio group versus HFD+Pio group.


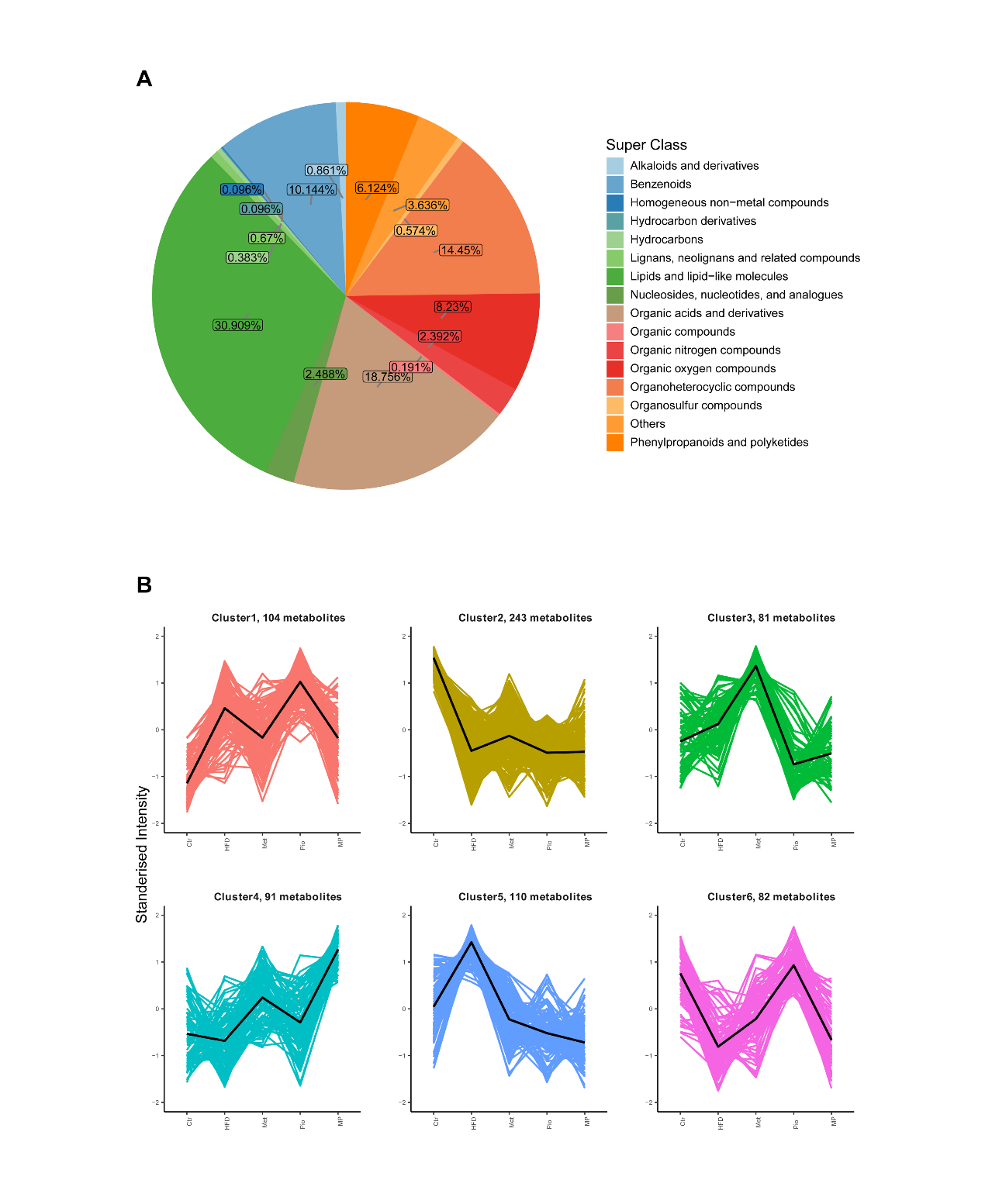


**Figure S5.** **K-means cluster analysis of the gut metabolites.** (A) Counts and class of metabolites detected using untargeted metabolomics; (B) K-means cluster analysis of the gut metabolites using the Hartigan–Wong algorithm. Ctr, standard control diet; HFD, high-fat diet; Met, high-fat diet treated with metformin; Pio, high-fat diet treated with pioglitazone; MP, high-fat diet treated with metformin and pioglitazone.


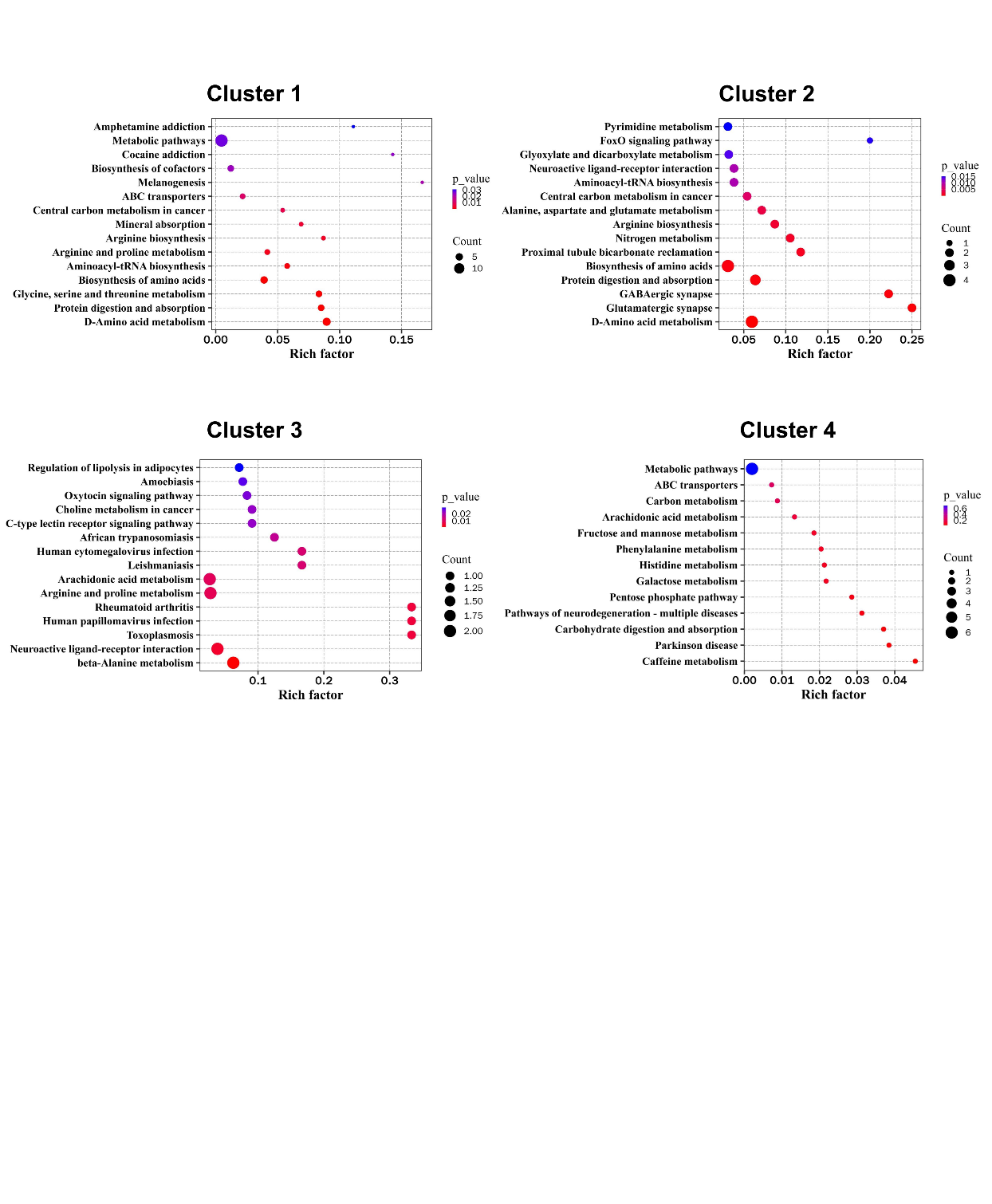


**Figure S6.** **KEGG enrichment analysis of altered gut metabolites in Clusters 1-4.**
